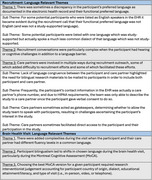# Recruitment and engagement of non‐English speaking participants in dementia research: Lessons from a pragmatic trial of dementia screening in primary care

**DOI:** 10.1002/alz70858_102113

**Published:** 2025-12-25

**Authors:** Leah Karliner, Alana Elop, Sherry Yam, Nana O'Donnell, Clarissa W Hsu, Judith Walsh, Tyler D Barrett, Deborah King, Mikael Anne Greenwood‐Hickman, Sascha Dublin, Deborah E Barnes

**Affiliations:** ^1^ University of California, San Francisco, San Francisco, CA, USA; ^2^ Kaiser Permanente Washington Health Research Institute, Seattle, WA, USA

## Abstract

**Background:**

To ensure new dementia research findings and treatments benefit all populations, researchers must include non‐English‐speaking patients in studies. However, in the U.S., these populations are often excluded due to the increased complexity created for research teams. This study describes challenges and learnings related to recruitment and engagement with non‐English speakers in a clinical cognitive assessment study.

**Method:**

The electronic health record Risk of Alzheimer's and Dementia Assessment Rule (eRADAR) trial is a pragmatic randomized controlled trial using an electronic health record (EHR)‐based algorithm to identify older adults at high risk of undiagnosed dementia and invite them to participate in a brain health assessment visit (BHV). Recruitment at one site included Chinese (Cantonese and Mandarin) and Spanish speakers alongside English speakers. We followed evidence‐based approaches to language translation, recruitment, and engagement of non‐English speakers in clinical research, including consultation with bilingual patient advisors. Research staff documented detailed comments about recruitment calls and BHVs in a tracking database. When enrollment was complete, three research staff reviewed all tracking comments (*n* = 574 participants) for language relevance and iteratively developed key themes in consultation with the larger research team. Supporting examples were summarized for each theme in a coding memo.

**Result:**

The team identified language‐relevant tracking comments for 83 participants: developing four recruitment outreach themes with six sub‐themes and three BHV‐specific themes (Table). Discrepancies between the EHR‐documented language and participants' preferred language impacted recruitment. For some participants, hearing and cognitive difficulties compounded the communication challenges across a language barrier. Care partners were crucial to recruitment, and we frequently needed to work with them to reach participants, which was not always successful. During BHVs, complexities arose from differences in care partner and participant language fluency, participant bilingualism, and selecting the appropriate version of the cognitive screening tool (Montreal Cognitive Assessment [MoCA]).

**Conclusion:**

In the eRADAR study, we followed evidence‐based approaches to inclusion of non‐English speakers in research and still uncovered important areas that required additional attention to the language needs of participants and their care partners. Other researchers should anticipate similar challenges and implement robust strategies to ensure inclusive and equitable participation in dementia studies.